# VenomKB, a new knowledge base for facilitating the validation of putative venom therapies

**DOI:** 10.1038/sdata.2015.65

**Published:** 2015-11-24

**Authors:** Joseph D. Romano, Nicholas P. Tatonetti

**Affiliations:** 1 Department of Biomedical Informatics, Columbia University Medical Center, New York, New York 10032, USA; 2 Department of Systems Biology, Columbia University Medical Center, New York, New York 10032, USA; 3 Department of Medicine, Columbia University Medical Center, New York, New York 10032, USA

**Keywords:** Translational research, Literature mining, Pharmacology, Toxicology

## Abstract

Animal venoms have been used for therapeutic purposes since the dawn of recorded history. Only a small fraction, however, have been tested for pharmaceutical utility. Modern computational methods enable the systematic exploration of novel therapeutic uses for venom compounds. Unfortunately, there is currently no comprehensive resource describing the clinical effects of venoms to support this computational analysis. We present VenomKB, a new publicly accessible knowledge base and website that aims to act as a repository for emerging and putative venom therapies. Presently, it consists of three database tables: (1) Manually curated records of putative venom therapies supported by scientific literature, (2) automatically parsed MEDLINE articles describing compounds that may be venom derived, and their effects on the human body, and (3) automatically retrieved records from the new Semantic Medline resource that describe the effects of venom compounds on mammalian anatomy. Data from VenomKB may be selectively retrieved in a variety of popular data formats, are open-source, and will be continually updated as venom therapies become better understood.

## Background & Summary

Venoms are substances that are secreted by animals for either defensive or offensive purposes and that have a toxic or harmful effect on other animals^1,2^. Different from poisons, which are harmful only when ingested, venoms must either be injected or applied topically to exert their effects. Although they were evolved to cause harm, a vast array of beneficial uses for specific venoms have been identified—some dating back thousands of years^3–5^. Many of these these traditional venom therapies have since been validated and new ones have been discovered^6^. Molecular studies conducted over the past century have revealed that venoms commonly consist of a complex cocktail of organic compounds^7^ and that the most active are usually peptide-based. For example, cone snails (genus *Conus*) have evolved complex venoms consisting of hundreds of unique compounds that rapidly paralyze prey before it is able to swim away. The most well characterized therapeutic use of cone snail venom-derived compounds is a peptide named ziconotide (from the species *C. magus*), an extremely potent analgesic used in cases of severe chronic pain^8^.

Furthermore, in recent decades it was discovered that individual venom-derived peptides usually have a highly specific molecular target, and a likewise specific molecular mechanism^6^. Combined with the facts that (a.) there are estimated more than 10 million uncharacterized venomous species of animals, and (b.) venoms are unique to each individual species (i.e., even closely related species within the same genus have completely unrelated venom profiles)^9,10^, animal venoms provide an unprecedented library of potentially beneficial pharmaceutically active compounds. However, the sheer number of compounds that require screening, validation, and characterization, along with the inherent toxicity of venoms, present a near intractable problem. In order to circumvent these challenges we propose the use of computational techniques to investigate both known and unknown venoms in a safe, cost-effective, and automated manner. A requisite component of these methods is the availability of a comprehensive resource describing the clinical effects of venom compounds stored in a computable format.

**Figure 1:**
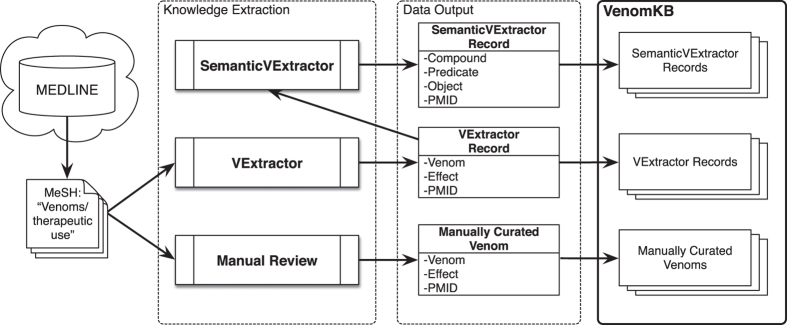
Schematic overview of building VenomKB. MeSH was used to identify a core set of relevant articles, which were then passed to three methods of knowledge extraction (manual review, the VExtractor algorithm, and the SemanticVExtractor algorithm). The outputs of these three methods were then collected and assembled as VenomKB.

**Figure 2:**
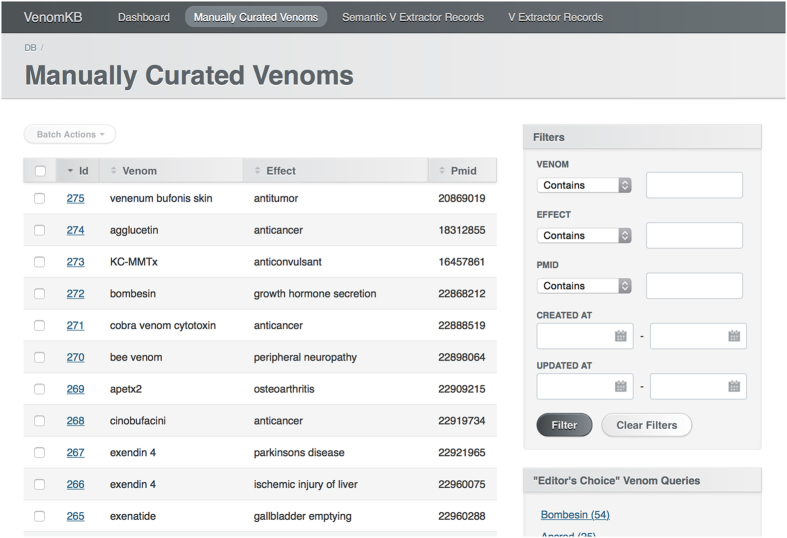
Screenshot of VenomKB’s knowledge base browsing interface. Image shows the first 8 records of the ‘Manually Curated Venoms’ table. The top bar has links to the knowledge base home page and each of the three current database tables. Search filters are in the frame entitled ‘Filters’ on the right side of the interface. Download links (for CSV, XML, and JSON format) and pagination functionality are located at the bottom of the page, out of range of the screenshot.

In this paper we introduce VenomKB—a new online knowledge base that is designed to facilitate the emergence of computational techniques to investigate therapeutic uses for venom compounds. At the time of writing, VenomKB consists of three database tables. The first is a manually curated list of putative and active (i.e., in clinical use) venom therapies. The second and third detail the outputs of two different algorithms (VExtractor and SemanticVExtractor) that were used to automatically extract (by natural language processing and knowledge discovery techniques) putative venom therapies in a corpus of abstracts from the scientific literature. A schematic outlining the processes of data collection and curation in building VenomKB is shown in Fig. 1. VenomKB is an open-source and publicly accessible resource for researchers and other individuals interested in venom therapeutics and may be accessed at the project’s official website (http://venomkb.tatonettilab.org). The website contains a tabular interface for searching, sorting, and viewing the different records in each database table, and data records of interest may be selectively downloaded in CSV, XML, and JSON formats, as desired. A screenshot of the database browsing interface is shown in Fig. 2. Additionally, a ‘frozen’ copy of the data as it exists at the time of this paper’s publication can be found on FigShare (see Data Citation 1, Data Citation 2 and Data Citation 3 for individual citations). The knowledge base currently contains 42,723 unique records.

The specific goals of VenomKB are twofold: (1) to make it easier to discover proposed or suggested venom therapies for a disease of interest (or vice-versa), and (2) to facilitate the identification of studies on established venom therapies in order to guide the study of newly discovered or newly classified venoms. Since the discipline of computational approaches for discovering venom therapies is emergent, we expect VenomKB to grow rapidly in the new future. We encourage interested users to monitor additions and changes to the knowledge base by viewing the website’s home page (venomkb.tatonettilab.org), which will be updated in the event of all major developments within both the knowledge base and the field of study as a whole.

## Methods

The MEDLINE biomedical literature repository contains 22,376,811 searchable titles and abstracts, as of its 2014 release^11^. We used this resource to extract all venom therapies, established or hypothetical. We found 5,117 relevant articles using the Medical Subject Heading (MeSH)^12^ ‘Venoms/therapeutic use’. We saved the abstracts in MEDLINE format—a text-based format that includes the article titles, abstracts, and important metadata records for each article. We then applied three separate methods for extracting data regarding putative venom therapies on these data—the first of these was manual review and curation of journal articles and the second and third were computational algorithms that automatically extracted relevant knowledge from the pre-filtered set of MEDLINE articles.

To manually curate the abstracts, we first randomized the order of the 5,117 journal articles (to avoid bias by only selecting articles from a short span of time) and selected the first 275 records that describe putative venom therapies. We skipped articles that were incorrectly tagged with the MeSH term ‘Venoms/therapeutic use’ or where the proposed venom therapy was unclear or subjectively deemed insignificant. We also ignored articles that described venom immunotherapy—a technique that involves reducing sensitivity to venoms (typically bee venoms) by administering small dosages of the venom over time in order to desensitize the immune response^13^. For abstracts that we determined contained valid putative venom therapies we recorded the data in the following format:

[venom|physologiceffect|PubMedID(PMID)]

In the first of the two automatic knowledge extraction methods, called VExtractor (see **Code Availability**; filename ‘vextractor.py’), we used the NCBO BioPortal Annator API to extract ontology terms from the text of the title and abstract for each of the 5,117 entries. We then filter the annotations by chosen ontologies and Unified Medical Language System (UMLS)^14^ semantic types that have a high likelihood of selecting venom compounds and physiological effects of the compounds on the human body. The ontologies and semantic types we used are documented in full in the repository listed in **Code Availability** (filename ‘vextractor_ontologies_and_semtypes.txt’). We selected these ontologies and semantic types based on knowledge of ontology contents and a trial-and-error process of manually altering the filtering strategy and observing if the algorithm was able to precisely identify venom compound names and physiological effects as was determined for five of the manually reviewed articles, selected randomly. Finally, VExtractor then sorts these terms into the appropriate data structures (see below) and returns them as output. The NCBO annotator can identify more than one venom compound and/or physiologic effect, so all were recorded for loading into the knowledge base. This application was run for each of the 5,117 originally identified journal articles as input—a task facilitated by the ability of VExtractor to accept a list of PMIDs and run the script for each one, returning the results as two comma separated value (CSV) text files, in the following format:

[PMID|venomcompound]

[PMID|physiologiceffect]

In this format, the results could be interpreted as a list (0 or more) of both potential venom compounds and effects. The outcome is greater flexibility in interpretation of the knowledge contained in a given article, at the expense of potentially losing resolution if multiple venom compounds—each with unique effects on the human body—are discussed in the same journal article. Finally, we combine these two lists using a Ruby script (see **Code Availability**; filename ‘make_vextractor_table.rb’) into a single list with records in the format:

[venomcompound|physiologiceffect|PMID]

We performed the second automated knowledge extraction method (a workflow we named ‘SemanticVExtractor’) using another new program named ‘SMDB_Search’ (see **Code Availability**; directory ‘smdb_search/’). SMDB_Search is a utility that connects to a local copy of the new Semantic MEDLINE (SemMedDB)^15^ resource—a database that enables searching MEDLINE by semantic concept rather than a traditional search query—and extracts semantic predicates for either a given list of PMIDs or a certain UMLS or Gene Ontology (GO) term. For the purposes of this study, we used all of the PMIDs that returned valid records in the VExtractor procedure (i.e., all for which at least one possible venom compound and at least one effect) as input for SMDB_Search. The output of SMDB_Search was a list of Java Script Object Notation (JSON) formatted data structures. It should be noted that instead of recording the output values as ‘potential venoms’ and ‘effects’ (as was done for the previous two knowledge extraction methods), we recorded them as ‘subject’ and ‘object’, since venoms and effects can occur in either order (e.g., ‘venom_x treats condition_y’ versus ‘condition_y treated_by venom_x’)—see Data Records for further explanation. In addition to the subjects and objects, we recorded the predicate phrase and the UMLS semantic types for each the subject and the object, to allow for more detailed analysis of data results in future additions to VenomKB. Finally, we used these semantic types to filter the output values of SemanticVExtractor—only data records with subject semantic types listed in Table 1 were retained, because those semantic types are the ones that logically may be assigned to venom compounds. Like with the VExtractor method, we designed SemanticVExtractor with the ability to return 0 or more data records for each journal article.

In order to remove a large number of the false positives identified by the two automated methods, we performed a manual review of the database contents, removing obviously erroneous entries. This included compounds that are not related to venom compounds (e.g., ‘insulin treats type-2 diabetes mellitus’), and uninformative/nonsensical entries (e.g., ‘medications treat patients’ or ‘venoms are venoms’).

### Code availability

All code to extract, process, and analyze data is available publicly, and is maintained on a GitHub repository (http://github.com/JDRomano2/venomkb_code). The code used for the VenomKB website is also available publicly on GitHub (http://github.com/JDRomano2/venomkb). All code is open source, and maintained under the GNU General Public License v2.

## Data Records

The results of the data collection methods described above are all available on the knowledge base website (http://venomkb.tatonettilab.org/), as well as in a public FigShare repository (see individual data citations below) for the purposes of data permanence and reproducibility of data integrity analyses that we have performed (see below, in ‘Technical Validation’). While the data records on FigShare are static, the content of the knowledge base itself will change over time as data records are validated/invalidated and as new knowledge extraction methods are developed for the emerging field of computationally-predicted venom therapies. To create the data files on FigShare, we exported the complete contents of the three relevant PostgreSQL tables as CSV-formatted files, where the first line of the file consists of the headers describing each data field, and each line thereafter represents a single data record. All of the tables include the following records: ‘id’ (a unique numerical identifier), ‘pmid’ (the PubMed identifier for an article supporting the data record), ‘created_at’ (the date and time at which the record was added to the database), and ‘updated_at’ (the date and time at which the record was most recently modified, which is identical to the contents of ‘created_at’ in many cases). Each of the three individual files is described below, with the addition of the other fields that are unique to each table.

The manually vetted putative venom therapies (‘Manually Curated Venoms’) are stored in a file named ‘manual_venoms.csv’ (Data Citation 1). A sample of the first three records is shown in Table 2. This table contains two unique fields: ‘venom’ and ‘effect’. ‘Venom’ is the name of the venom compound. These names may or may not be a trade name, a compound name, or some other name, but they reflect the name used in the associated journal article. It should be noted that this is not an arbitrary design decision—since there is no standardized naming format or classification system for venom components (e.g., the compound EMD 121974—a modified snake venom protein—is almost ubiquitously referred to by the trade name Cilengitide), the most methodical approach is simply to preserve the name(s) given by the author of the journal article. ‘Effect’ is the primary purported physiologic, molecular, or phenotypic effect or target of the venom. However, this is not explicitly qualified—for example, a venom compound that is reported as ‘effect’ being ‘Parkinson’s disease’ likely intends to mean that the venom treats Parkinson’s disease, not that it causes the disease. Although this introduces some ambiguity into the database, it was a design choice made to facilitate easy searching for diseases and molecular targets via the web interface.

The data for the first automated knowledge extraction algorithm—which utilizes the NCBO Annotator API; named VExtractor—is contained in the file named ‘vextractor.csv’ (Data Citation 2). A sample of the first three records is shown in Table 3. Aside from the common fields mentioned above, these data records have two additional fields: ‘venom’ and ‘effect’. Like with the other methods, ‘venom’ describes the venom or venom component being discussed. Since these terms were automatically extracted and standardized to the terms contained in the UMLS, the naming scheme is more consistent than in the Manually Curated Venoms table. ‘Effect’ is similar to the equivalent field in Manually Curated Venoms, but it is more commonly a disease or an observable physiological effect rather than a molecular mode of action or molecular target. Likewise, although the effect is often listed as a disease name, it should usually be interpreted as *treating* that disease rather than causing it. This should, however, be done with regard to context: venom compounds in many situations may in fact be the cause of particular generalizable diseases (e.g., pancreatitis as a result of *Tityus trinitatis* scorpion envenomation^16^). For this reason, we urge users to refer to the supplied PubMed IDs when looking at individual VenomKB records. The label for the column was not chosen to be ‘treats’ because the field does not always describe a treatment. If there is any ambiguity in a data record of interest, it is strongly recommended to view the cited PubMed article to determine the exact context of the therapeutic effect of the venom.

**Figure 3:**
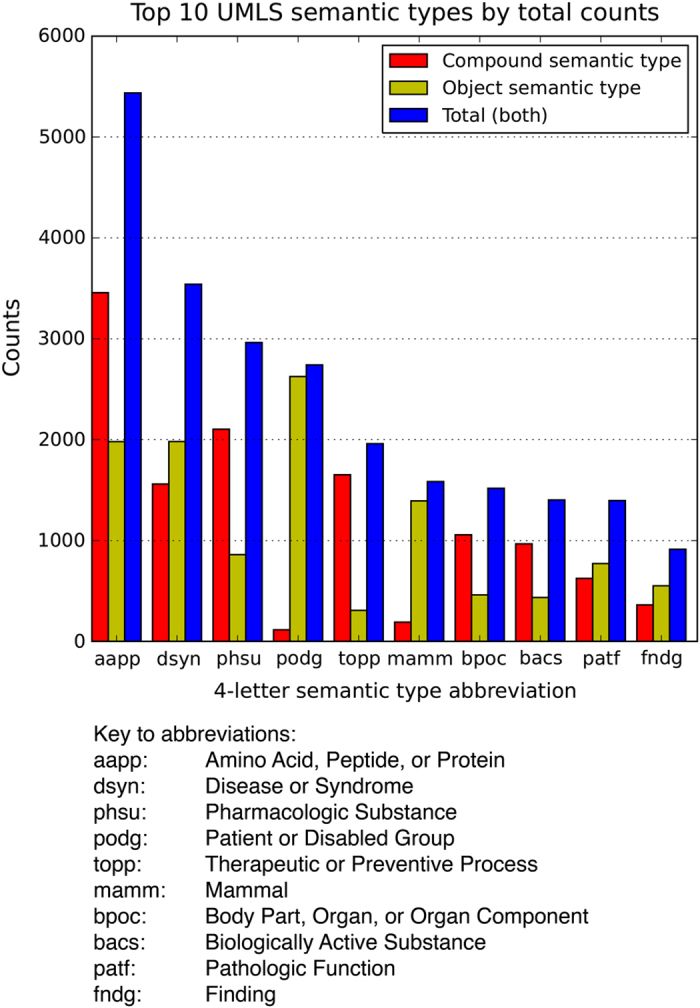
The top 10 most frequent UMLS semantic types represented in the SemanticVExtractor data output, graphed by total counts. Shown separately are counts for compound semantic types, object semantic types, and the sum of both, each plotted in separate colors as indicated by the figure legend. The x-axis lists each of the top 10 UMLS semantic types by their 4-letter abbreviation, for ease of plotting. Each abbreviation is defined beneath the plot, in descending order of frequency.

Formatted output from the second automated method—using the SMDB_Search utility; named SemanticVExtractor—is contained in a file named ‘semantic_vextractor.csv’ (Data Citation 3). A sample of the first three records is shown in Table 4. Unlike the prior database tables, this one contains three fields of interest: ‘compound’, ‘predicate’, and ‘object’. These three fields describe the three components of a predication stored in the SemMedDB database—a subject, a predicate, and an object. A predication describes a relationship between two entities (the subject and the object), and its predicate defines the type of relationship. The order ‘*subject*|*predicate*|*object*’ has the advantage of being similar to the structure of an English language sentence, so the semantic concept underlying the predication can be easily read by a human. For example, if the predication is ‘caerulein|augments|pancreatic juice secretion’, it is easily understood as equivalent to the phrase, ‘The (venom-derived) compound named caerulein augments the secretion of pancreatic juice.’ In this context, the subject of the predication is always a chemical compound, so the ‘subject’ field of SemanticVExtractor output was renamed to ‘compound’ upon loading into the knowledge base. However, the venom component being referred to is not always the subject—it could also be the object of the predication. For instance, one of the predications in this table could be ‘*compound ‘X’*|*inhibited_by*|*bombesin*’. This predication describes the effect of bombesin on compound ‘X’, yet bombesin is the object of the predication. In this table, ‘compounds’ and ‘objects’ are always either UMLS terms or GO terms, and ‘predicates’ are the predicates that are contained within the SemMedDB database (specifically contained in the ‘PREDICATION’ table of the database). A parallel bar chart of the 10 most frequent semantic types in Semantic VExtractor is shown in Fig. 3.

To improve the utility of VenomKB beyond that of a purely static knowledge resource, individual web pages describing the data records contain links to their cited PubMed articles, as well as links to search queries for compounds and other terms on a number of external databases/ontologies. Since there is no structured terminology or naming scheme for venoms and/or venom derived compounds, we cannot guarantee that all records in VenomKB will return useful search results—this is something that we intend to improve upon in the future by creating a hierarchical terminology of venoms that can be used to standardize the contents of VenomKB, and generate cross-mappings to other knowledge resources regardless of synonym variation.

## Technical Validation

The manually reviewed and curated list of putative venom therapies was considered the ‘gold standard’ against which the two automated methods of knowledge extraction were validated. Validation was performed to determine the ability of the two automated methods to identify venom compounds and their purported effects on the human body. It was assumed that the precision of the two automated methods would be low, since there is no UMLS semantic type or other unique identifier with which venom compounds are annotated in a consistent manner in the scientific literature. As a result, many of the identified compounds are not venoms at all, but belong to the same UMLS semantic types as venoms and venom components. However, the two algorithms were designed to be highly sensitive. In essence, we expected to see a high occurrence of false positives but a substantially lower occurrence of false negatives.

In order to determine percent recall of the two algorithms, we selected 100 records at random from the table of manually reviewed venom therapies. For each of those selected data records, we then manually recorded whether the same venom compound and effect were identified by each of the two algorithms for the MEDLINE article associated with the respective PMID. For this measurement, VExtractor exhibited a 76% recall with respect to the gold standard, and SemanticVExtractor exhibited a 67% recall. Additionally, we recorded whether the ‘*venom*|*effect*’ pair was found in any record, regardless of PMID. For this second measurement (where the ‘PMID’ field was disregarded), VExtractor had a recall of 89% (a change of +13%) and SemanticVExtractor had a recall of 84% (a change of +17%). These data support the conclusion that the two algorithms have a relatively high degree of sensitivity for correctly extracting venoms and their purported effects, and the false negatives (‘*venom*|*effect*’ pairs not identified by one of the two algorithms) are substantially offset by the ability of the algorithms to identify equivalent ‘*venom*|*effect*’ pairs elsewhere in the scientific literature. We calculated the specificity of each of the two algorithms by selecting 100 random records and determining whether those records describe a venom or a venom compound, and also whether they describe a physiologic target or effect of that venom compound. Prior to pruning obvious false positives from each of the two database tables, VExtractor demonstrated a precision rate of 66%, and SemanticVExtractor demonstrated a precision rate of 52%. After pruning false positives, we resampled the two database tables and recomputed precision. Each of the two values improved substantially: VExtractor demonstrated a new precision rate of 82% (a change of +16%), and Semantic VExtractor demonstrated a precision rate of 80% (a change of +28%), empirically demonstrating the value of manually filtering ‘bad values’. These values (both recall and precision) are shown in Table 5, and the specific data records used to conduct the validation are available on FigShare (Data Citation 4).

Although these rates for precision are relatively high for a novel knowledge discovery pipeline, they do raise the question of how to minimize false positives in a maintainable fashion, rather than via manual review and culling of erroneous records within very large database tables. As mentioned below (in Usage Notes), VenomKB allows for users to ‘flag’ individual records for removal. This method of ‘crowd sourcing’ the removal of erroneous records will continue to improve in its robustness as VenomKB gains content and new users.

False negatives are another important concept to consider. Our method for identifying relevant PubMed articles involved searching for the MeSH term ‘Venoms/therapeutic use’, but since MeSH terms are manually curated annotations, there is no way to ensure full coverage of relevant articles. Furthermore, a lack of structured terminological resources for studying venoms and venom components makes more complex methods of knowledge retrieval (e.g., using alternative machine learning techniques that incorporate semantic knowledge of venom compounds) nearly impossible. To this end, we are planning a follow-up study that involves the creation of an ontology for venoms and their contained compounds, as well as synthetic derivatives that are already used therapeutically. After creating the ontology, we should be able to devise novel methods for identifying false negatives—those records erroneously omitted from the database due to a lack of complete MeSH term annotation.

## Usage Notes

Any internet enabled device using a modern web browser should be able to access the knowledge base and download data from both the knowledge base itself (http://venomkb.tatonettilab.org) and from the FigShare repository (Data Citation 1, Data Citation 2, and Data Citation 3). No user accounts are necessary to access or download the data records, but a number of community editing and contribution features do require users to create a private profile. Users have the ability to add or edit records on the manually-curated portion of the knowledge base. Deletion privileges are not publicly available, in order to prevent abuse. However, if a user feels that a particular record was included erroneously, there is a button on the data record’s page that allows the user to ‘flag’ the record for review by site administrators. Once flagged, administrators are notified, after which they decide whether to remove the item or not. Users can see on the index page whether individual items have been flagged or not. Furthermore, users may contribute to a ‘comment’ thread on each data record, given that they have logged in to an account. Comment threads are visible on the pages for each individual database record. Major changes to the knowledge base are announced on the knowledge base website when they occur. As mentioned previously, data records may be selected and downloaded in one of three software formats: CSV, XML, and JSON. Although these may be manipulated and analyzed by most modern programming languages and data analysis software packages, we performed technical validation of the data sets using the standard libraries of the Python and Ruby programming languages. Intermediary data files (prior to loading into a relational database) were structured as to make them ‘self documenting’ (i.e., key-value pairs include descriptive key labels). A GitHub repository with all of the scripts used to analyze and process the data is linked to in the **Code Availability** section. Within the knowledge base, numerical identifiers for individual records were assigned arbitrarily based upon the order in which they were added to the database.

As mentioned previously, VenomKB will change and grow as new records are added. In particular, we plan to expand the ‘manually curated venoms’ database by identifying additional relevant MeSH terms that may also refer to therapeutic uses of venoms (aside from ‘Venoms/therapeutic use’). Furthermore, we plan to closely monitor new studies regarding novel venom therapies, adding them to the knowledge base as we come across them.

Since VenomKB is intended to grow into a collaborative, public resource on computational analysis and prediction of putative venom therapies, we encourage suggestions and comments regarding new additions and revisions. Up-to-date contact information can be found from the homepage of the knowledge base website, or alternatively, readers can contact the corresponding author for this study as listed below.

A final note to users regards data records that appear to be irrelevant at first glance, yet actually do describe a property of a venom compound being used for therapeutic purposes. For example, consider entries in the VExtractor database for PMID 22098810. The database contains 4 entries for this PMID, all referring to a venom compound named ‘hypoglycemic agent’, which treats ‘obesity’. Upon inspecting the journal article referenced by this PMID, it can be seen that the hypoglycemic agent in question is actually the venom compound ‘exenatide’, which does treat both type-2 diabetes mellitus and obesity^17^. As mentioned previously, we plan to build a structured terminology for venom compounds that can be used to resolve relatively uninformative descriptors (such as ‘hypoglycemic agent’) into their actual specific venom compound names, but since such a resource currently does not exist, we suggest that users follow links to the PubMed pages to validate the compound(s) themselves.

## Additional Information

**How to cite this article:** Romano, J. D. and Tatonetti, N. P. VenomKB, a new knowledge base for facilitating the validation of putative venom therapies. *Sci. Data* 2:150065 doi: 10.1038/sdata.2015.65 (2015).

## Supplementary Material



## Figures and Tables

**Table 1:** UMLS Semantic Types for filtering SemanticVExtractor output

**UMLS Semantic Type**	**4-letter abbreviation**
Amino Acid, Peptide, or Protein	aapp
Amino Acid Sequence	amas
Biologically Active Substance	bacs
Body Substance	bdsu
Chemical	chem
Chemical Viewed Functionally	chvf
Chemical Viewed Structurally	chvs
Clinical Drug	clnd
Eicosanoid	eico
Enzyme	enzy
Hazardous or Poisonous Substance	hops
Hormone	horm
Immunologic Factor	imft
Nucleic Acid, Nucleoside, or Nucleotide	nnon
Neuroreactive Substance or Biogenic Amine	nsba
Organophosphorus Compound	opco
Pharmacologic Substance	phsu
Substance	sbst
All predications returned by SMDB_Search within the SemanticVExtractor algorithm were filtered by the predication’s subject semantic type. In order for a data record to be retained, its subject (named ‘compound’ in the final knowledge base table) semantic type must be one of the UMLS semantic types listed in this table. These particular semantic types were selected because a venom compound may be grouped into any one of them.	

**Table 2:** Sample data from ‘Manually Reviewed Venoms’ database table.

**id**	**venom**	**effect**	**pmid**
1	bombesin	gastric secretion	11996
2	ancrod	claudication	66429
3	ancrod	deep vein thrombosis	80632
…	…	…	…
*n*	*[venom_n]*	*[effect_n]*	*[pmid_n]*

**Table 3:** Sample data from ‘VExtractor’ database table.

**id**	**venom**	**effect**	**pmid**
1	ceruletide	tachyphylaxis	11996
2	ceruletide	gastric secretion	11996
3	ceruletide	pancreatitis	2717605
…	…	…	…
*n*	*[venom_n]*	*[effect_n]*	*[pmid_n]*

**Table 4:** Sample data from ‘SemanticVExtractor’ database table.

**id**	**compound**	**predicate**	**object**	**pmid**
1	bombesin	isa	tetradecapeptide	11996
2	bombesin	augments	gastric	11996
3	caerulein	affects	acidification	11996
…	…	…	…	…
*n*	*[compound_n]*	*[predicate_n]*	*[object_n]*	*[pmid_n]*

**Table 5:** Summary of technical validation results

**Algorithm Name**	**Number of records**	**Recall (PMID-sensitive)**	**Recall (PMIDs disregarded)**	**Precision**
VExtractor	35,240	76%	89%	82%
SemanticVExtractor	7,208	66%	84%	80%
Percent recall rates of the ‘VExtractor’ and ‘SemanticVExtractor’ algorithms as compared to the gold standard (manually reviewed venoms), using 100 randomly selected records from the ‘Manually Reviewed Venoms’ table. Reported precision was computed after manually pruning obvious false positives.				
